# Information and Communication Technology-Powered Diabetes Self-Management Systems in China: A Study Evaluating the Features and Requirements of Apps and Patents

**DOI:** 10.2196/diabetes.4475

**Published:** 2016-04-06

**Authors:** Ying Li, Jin Tan, Bozhi Shi, Xiaolian Duan, Daidi Zhong, Xiaoling Li, Jianning Qu

**Affiliations:** 1 Key Laboratory of Biorcheological Science and Technology, Ministry of Education Bioengineering College Chongqing University Chongqing China; 2 Chongqing Academy of Science & Technology Chongqing China; 3 The Second Affiliated Hospital of Chongqing Medical University Chongqing China

**Keywords:** apps, diabetes, information and communication technology, self-monitoring of blood glucose, patents

## Abstract

**Background:**

For patients with diabetes, the self-monitoring of blood glucose (SMBG) is a recommended way of controlling the blood glucose level. By leveraging the modern information and communication technology (ICT) and the corresponding infrastructure, engineers nowadays are able to merge the SMBG activities into daily life and to dramatically reduce patient’s burden. Such type of ICT-powered SMBG had already been marketed in the United States and the European Union for a decade, but was introduced into the Chinese market only in recent years. Although there is no doubt about the general need for such type of SMBG in the Chinese market, how it could be adapted to the local technical and operational environment is still an open question.

**Objective:**

Our overall goal is to understand the local requirements and the current status of deploying ICT-powered SMBG to the Chinese market. In particular, we aim to analyze existing domestic SMBG mobile apps and relevant domestic patents to identify their various aspects, including the common functionalities, innovative feature, defects, conformance to standards, prospects, etc. In the long run, we hope the outcome of this study could help the decision making on how to properly adapt ICT-powered SMBG to the Chinese market.

**Methods:**

We identified 289 apps. After exclusion of irrelevant apps, 78 apps remained. These were downloaded and analyzed. A total of 8070 patents related to glucose were identified from patent database. Irrelevant materials and duplicates were excluded, following which 39 patents were parsed to extract the important features. These apps and patents were further compared with the corresponding requirements derived from relevant clinical guidelines and data standards.

**Results:**

The most common features of studied apps were blood health data recording, notification, and decision supporting. The most common features of studied patents included mobile terminal, server, and decision supporting. The main difference between patents and apps is that the patents had 2 specific features, namely, interface to the hospital information system and recording personal information, which were not mentioned in the app. The other major finding is that, in general, in terms of the components of the features, although the features identified in both apps and patents conform to the requirements of the relevant clinical guidelines and data standards, upon looking into the details, gaps exist between the features of the identified apps and patents and the relevant clinical guidelines and data standards. In addition, the social media feature that the apps and patents have is not included in the standard requirements list.

**Conclusions:**

The development of Chinese SMBG mobile apps and relevant patents is still in the primitive stage. Although the functionalities of most apps and patents can meet the basic requirements of SMBG, gaps have been identified when comparing the functionalities provided by apps and patents with the requirements necessitated by the standards. One of the most important gaps is that only a small portion of the studied apps provides the automatic data transmission and exchange feature, which may hamper the overall performance. The clinical guidelines can thus be further developed to leverage new features provided by ICT-powered SMBG apps (eg, the social media feature, which may help to improve the social intervention of patients with diabetes).

## Introduction

### Self-Monitoring of Blood Glucose

According to World Health Organization data statistics, in 2014, the global prevalence of diabetes was estimated to be 9% among adults [1]. In most developed countries, diabetes is the fourth highest fatal disease, whereas in developing countries, the number of people living with diabetes is on the rise [2]. Being a developing country, China has the largest population of diabetics in the world [3,4]. With the increasing prevalence of diabetes, various diabetes-related complications have become the main reason for disability or death in these patients [5].

Results of clinical studies have shown that controlling glucose level based on a predefined rule can reduce the incidence and development of chronic complications of diabetes, but whether glucose level can remain stable in an allowed range in daily life depends on diabetes self-management [6,7]. The self-monitoring of blood glucose (SMBG), as a recommended diabetes self-management tool, plays an important role in the self-management of diabetes. It can provide real-time glucose information and timely intervention that can help patients maintain normal blood sugar levels. The demonstrated clinical value of SMBG for patients with diabetes has been widely recognized by stakeholders. Accordingly, the International Diabetes Federation [8], the American Diabetes Association [9], and the National Institute for Clinical Excellence [10] have stressed that SMBG is an integral part of a comprehensive diabetes management, and they recommend that all patients should practice SMBG. However, the data from SMBG can significantly affect physician’s decision making because, in their opinion, the influence of the SMBG data for treatment decisions is equal or greater than the glycosylated hemoglobin (HbA1c) levels [11-15]. The guidelines [16] proposed by the Ministry of Health (MoH) also promoted various subtypes of SMBG scheme applicable to patients with diabetes.

### Information and Communication Technology

In 2014, there were about 1.5 billion mobile device users worldwide. A year ago, this number was only 1.1 billion. A previous report [17] indicated that P.R. China had the largest amount of mobile phone users in 2013 and the number is still increasing. The information and communication technology (ICT) [18], characterized by multimedialization, popularization, diversity, personalization, and globalization [19], is an attractive platform for health promotion and disease management interventions. The same applies to diabetes self-management as well.

The Chinese government acts positively in deploying ICT in the health care domain, as it has already issued several regulatory policies in recent years, such as the “Twelfth Five (2011-2015)” National Strategic Emerging Industry Development Plan [20], the Biomedical industry development plan [21], and the Opinions on Promoting the Development of Health Services Industry [22]. The top policymakers in the country are pushing stakeholders to develop portable health data-collection devices, with ability to connect to the Internet (eg, via Wi-Fi or mobile Internet), and to increase the deployment of automatic and intelligent health information service.

### SMBG System Powered by ICT

**Figure 1:**
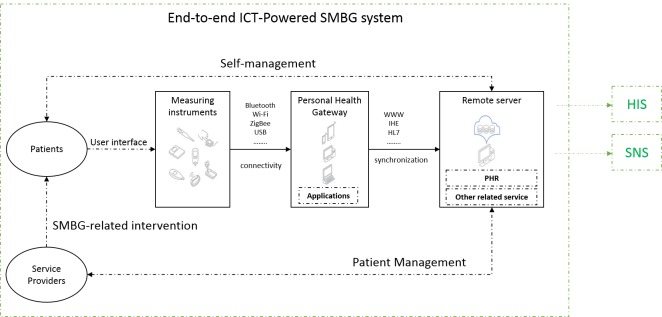
The user flow of the SMBG practice based on an end-to-end system.

When practicing SMBG, patients normally monitor and manage their glucose level by themselves at home. Such an activity may benefit from an ICT-powered SMBG system, which allows patients to transmit their data to a service provider via an end-to-end data channel. In most cases, such a system is composed of one or more measuring instruments (eg, a glucose meter), a gateway device (eg, a mobile device), and a remote server. Patients can collect their glucose measurements and other related health data with glucose meter and other instruments. The common devices used here are blood glucose monitor (BGM) and continuous glucose monitor (CGM). The remote server contains personal health record (PHR) and other related services. The established connectivity between measuring instruments and personal health gateway, and the connectivity between gateways and remote server, together populate an end-to-end data channel. Through this channel, the collected data can be transmitted from patients to service providers via uplink, and the instructions from service providers can be sent to patients via downlink. This allows patients and service providers to access the health data any time. Service providers can provide further appropriate interventions to patients based on certain data-driven strategy. Moreover, the external partners contain the hospital information system (HIS), the social network site, etc. Figure 1 shows a general architecture of such ICT-powered end-to-end system. It is a simplified version of the architecture described in Continua Design Guidelines [23], which has been widely adopted by vendors in the European Union and the United States. With the rapid development of ICT, there are many mobile apps about disease management in developed countries [24-26], especially about diabetes self-management [27].

The SMBG powered by the aforementioned system can effectively assist patients with lifestyle changes and reduce patients’ reliance on doctors. In addition, it allows the service providers to acquire the information about patients in a timely manner, to promote patients’ self-management and to generate revenue for primary care stations. This technology can not only set patients free, but can also reduce the financial burden of hospitalization and medical resource consumption. Furthermore, previous studies have shown that such system may lead to positive clinical effectiveness. Hua [28] and Yan [29] carried out experiments using the ICT-powered SMBG system, and the experimental group outperformed the control group in terms of the clinical efficiency of diabetes treatment, the stability of controlled blood glucose level, and the efficacy of drug usage. Last, but not least, with the help of the aforementioned system, service providers now have great potential to conduct personalized data mining and analysis based on the acquired health care information, and thus are able to provide various customized treatment solutions [30].

### Contents and Objectives

The ICT-powered SMBG had already been marketed in the United States and the European Union for a decade, but was introduced into the Chinese market only in recent years. Although there is no doubt about the general need for such a type of SMBG in the Chinese market, how it could be adapted to the local technical and operational environment is still an open question.

Our overall goal is to understand the local requirements and the current status of deploying ICT-powered SMBG to the Chinese market. In particular, we aim to analyze existing domestic SMBG mobile apps and relevant domestic patents to identify their various aspects, including the common functionalities, innovative feature, defects, conformance of standards, prospects, etc. In the long run, we hope that the outcome of this study could help with the decision making on how to properly adapt ICT-powered SMBG to the Chinese market.

Although the end-to-end ICT-powered SMBG system is the target of this study, we employed mobile apps as one of our data resources. From the architecture presented in Figure 1, it can be seen that the mobile app is a key component. Its features may well reflect the features of the entire system. Moreover, it is much easier to acquire metadata of mobile apps, than those of the end-to-end system. This can be achieved by searching the app stores or Internet.

## Methods

This section describes how the apps, the patents, and the standards were identified, and what selection strategy had been applied in this study. It also shows how the studied apps and patents were evaluated and analyzed.

### Apps and Patents Selection Strategy

In this study, the search resources are online stores for mobile apps and Chinese national patents database [31]. We searched for diabetes-related apps from globally available app stores including Google Play Store, iTunes, BlackBerry World, Windows Phone, and Nokia’s store. The domestic app stores in PR China such as Huawei Store [32] and Xiaomi Store [33] were also searched. We identified 289 apps and 8070 patents from app store and patents database, respectively, using the following keywords: “glucose,” “diabetes,” and “chronic disease management.” Among the search result of apps, in particular, the Google Android market occupies the largest portion (n=195), followed by the Apple iOS (n=94); no results were retrieved from the Blackberry and Windows Phone store.

In the next step, we eliminated general health and lifestyle apps and game apps, because they were not relevant to the SMBG practice (eg, the calorie calculator, glucose calculator). We also excluded apps that are not designed for the Chinese market and are not in Chinese. This is to ensure that all identified apps are available in the Chinese market and that they present the information in Chinese. Finally, we excluded apps that are not related to recording glucose levels for patients with diabetes.

**Figure 2:**
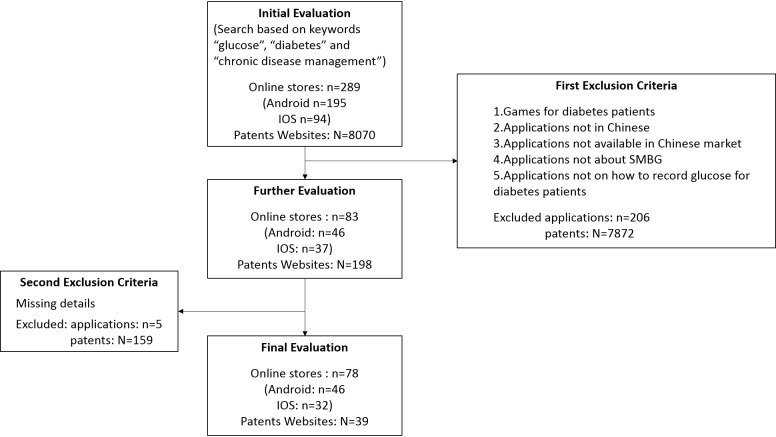
The flowchart of selection strategy.

Thus, after the first round of selection, a total of 83 apps and 198 patents remained. The second round of selection aimed at identifying apps on the management of diabetes without much missing details. A total of 78 apps (Android: n=46 and iOS: n=32) and 39 patents were selected. Figure 2 shows the inclusion and exclusion criteria applied in this study.

### Standards Selection Strategy

The application of various standards plays an important role in regulating the product design, product usage, and clinical practices. Therefore, conformance to the requirements imposed by the respective standard is essential to ensure that the ICT-powered SMBG system implemented is both functionally and clinically suitable. Besides, adherence to necessary standards is also a necessary condition for such implementation to become scalable and widely deployable.

To meet these rationales, 2 types of standards are considered in this study: the clinical guidelines and the data standards. The former ensures that the ICT-powered SMBG implementations are capable of meeting the service provider’s operational and clinical needs, and the latter ensures that multiple technical elements (devices and services) work together in an interoperable way. In the context of any end-to-end system having an architecture similar to Figure 1, the so-called data standards actually refer to a family of technical standards, including data exchange protocol, wired or wireless transport, data dictionary, nomenclature code, domain information model, device profile, service configuration, testing procedure, etc. However, providing their technical detail is out of the scope of this article.

To identify relevant data standards, we searched a database of national standard [34], and 3 databases of ministry-level standards, including the MoH [35], Ministry of Industry and Information Technology [36], and China Food and Drug Administration (CFDA) [37] databases. The only reference we found is the *basic dataset of disease management*, which was published by the MoH in 2012 [38]. However, this standard is purely a data dictionary specifically designed for capturing intrahospital information exchange (eg, HIS) data. The content of the standard alone is not able to support all the operations and data flows generated by an ICT-powered end-to-end system.

Therefore, as a backup, we employed the following 2 international data standards, whose scopes are similar to what we are looking for, as the baseline for this study: IEEE 11073-10417-2015 (BGM) [39] and IEEE 11073-10425-2014 (CGM) [40], both of which were published by the Institute of Electrical and Electronics Engineers (IEEE) in 2015 and 2014, respectively, and are now being adopted by the International Organization for Standardization.

For the clinical guideline, we identified 2 relevant guidelines [41-42] published by the Chinese Diabetes Society in 2011 and 2012, respectively.

### Evaluation and Analysis Method

**Figure 3:**
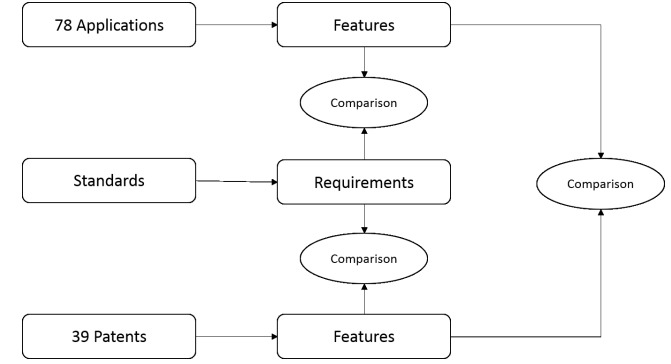
The flowchart of analysis method.

After obtaining the apps and patents, we summarized the features of the apps and patents, and also extracted relevant requirements from standards. The features of these apps and patents are listed in Multimedia Appendices 1 and . The requirements exacted from standards are listed in the “Data Standards” section, and the following comparisons are presented: (1) comparison between the features of apps and the features of patents; (2) comparison between the features of apps and the requirements of standards; (3) comparison between the features of patents and the requirements of standards. The flowchart of the analysis method we employed is shown in Figure 3.

## Results

### Data Standards

**Figure 4:**
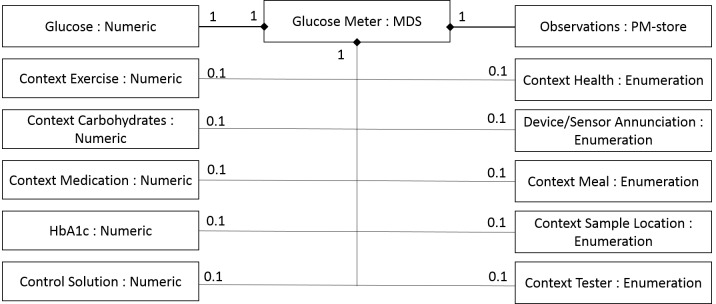
The domain information model in IEEE Std.11073-10417-2015.

**Figure 5:**
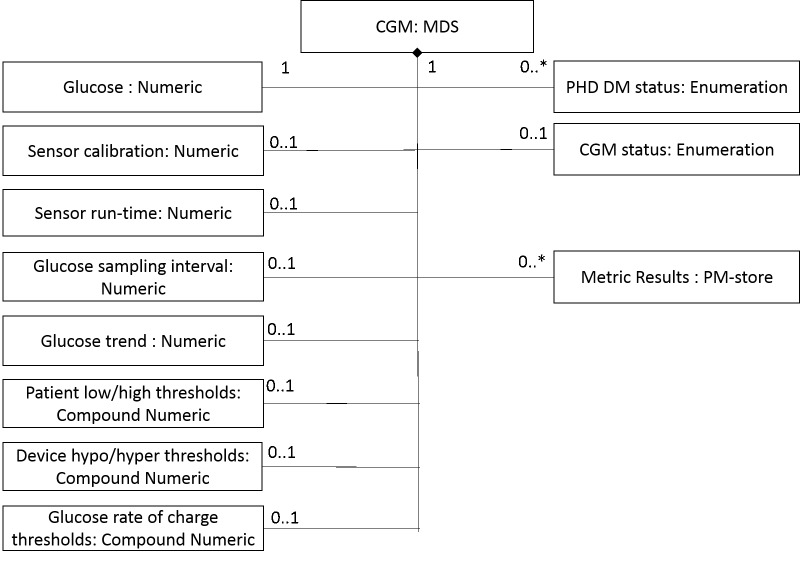
The domain information model in IEEE Std.11073-10425-2014.

The data standards we used in this study were IEEE 11073-10417-2015 (BGM) and IEEE 11073-10425-2014 (CGM). Within these 2 data standards, the key measurements, data format, nomenclature codes, data exchange method, device parameters, and device profiles are defined to ensure the interoperability between BGM/CGM devices, gateway devices, and backend servers. Figures 4 and 5 show the domain information model of the 2 standards. Such models may help us to deduce the data-related requirements for SMBG apps, and such requirements come from industry consensus built from the standard development process.

The aforementioned basic dataset of disease management only contains basic glucose level and HbA1c measurement, whereas the 2 IEEE standards contain far more measurements and contextual information, which may better satisfy the needs of SMBG. Such supplementary information can be summarized and categorized as follows:

1. *Direct measurements:* glucose, blood pressure;

2. *Indirect measurement or manual input:* diet, medications, exercise, symptoms, tester, sample locations; and

3. *Instrument information:* interval, author code, alert, calibrations, device status, and solution.

### Clinical Guidelines

The clinical guidelines used in this study were the Chinese Glucose Monitoring Clinical Guideline and the Chinese Continuous Glucose Monitoring Clinical Guideline. These 2 clinical guidelines define the data that service providers need to know from the patients in SMBG, and provide a standard of glucose monitoring for patients and doctors, including measure counts, exact measuring time, data format, and calculated statistical information of CGM, which is the most useful data for accurate diagnosis. Such standardized presentation of SMBG data can largely help clinicians to understand the conditions of patients with diabetes.

The requirements suggested by such clinical guidelines, either directly or indirectly, can be summarized and categorized as follows:

1. *Accuracy-related information:* accuracy of BGM device, contextual factors of BGM, and accuracy of CGM;

2. *Glucose-related statistics:* frequency and time point of glucose monitoring, mean value, standard deviation, volatility, and absolute difference;

3. *Other necessary context information:* diet, exercise, weight, blood pressure, medications, and symptoms.

4. Data evaluation and analysis method;

5. Decision-supporting information;

6. Diabetes-related educational material;

7. PHR.

### Functionality of the End-to-End SMBG System Suggested by Referenced Standards

By jointly considering the data standards and the clinical guidelines, we have identified the following requirements on functionalities:

1. *Self-test:* This includes blood glucose, blood pressure, weight, diet status, record of exercise, record of medications, record of symptoms;

2. Diabetes-related education material;

3. Personalized context-based notification and alert;

4. Decision-supporting information;

5. Data synchronization to PHR; and

6. Data evaluation and analysis method.

The aforementioned functionalities are further elaborated in Table 1. Although these functionalities are mutually exclusive, they have the potential to work as an integrated solution to support SMBG practices. For example, the user can log into the personal portal to view the records of glucose level, blood pressure, weight, diet, exercise, medication, and symptoms to evaluate how these factors are inter-related or how they may affect his/her blood glucose condition.

**Table 1:** Descriptions of the functionalities implied by standards.

No	Function	Input	Description	Source^a,b^
1.1	Blood glucose	Blood glucose values timestamp	User enters values and can view graphs, with low, high, and normal ranges well demarcated.	D/C
1.2	Blood pressure	Blood pressure timestamp	User enters values and can view graphs, with low, high, and normal ranges well demarcated.	D/C
1.3	Weight	Weight timestamp	User enters values and can view graphs.	D/C
1.4	Diet status	Food eaten, carbohydrate timestamp	User enters values and manually selects the type and amount of food.	D/C
1.5	Record of exercise	Exercise type, intensity, duration	User enters values and manually selects the type and intensity of exercise.	D/C
1.6	Record of medications	Medications type, amount, timestamp	User enters values and manually selects the type and amount of drugs.	D/C
1.7	Record of symptoms	Symptoms timestamp	User enters values and manually enters daily physical condition.	C
2	Diabetes-related education material	Tips, feedback, diabetes health information	Most apps linked to another app for educational material or used Web links. Some had decision support and personalized tips and feedback.	C
3	Personalized context-based notification and alert	Injection reminders, medication reminders, measuring reminder	User enters reminders manually, and also receives reminders for postprandial testing. Some had automatic tailored alerts.	D
4	Decision-supporting information	Emails between doctors and patients	Data can be shared between doctors and patients, and doctors can give remote guidance.	C
5	Data synchronization to personal health record (PHR)	PHR	Synchronization with personal health systems, data can be shared and stored in the cloud platform for easy access.	C
6	Data evaluation and analysis method	The signs data	The data can be presented in a graph to show the effects of data to blood glucose for doctors to determine.	C

^a^C: Functionalities from the clinical guidelines.

^b^D: Functionalities from the data standards.

### Mobile Apps

A total of 78 identified apps have been downloaded, and their functionalities were studied. Most of these apps provide similar functionalities, such as blood health data recording, notification, and offer decision-making support. Most functionalities listed in Table 1 can also be found in the studied apps.

Among them, the blood glucose recording is the most common functionality, which is obvious. A self-management portal allowing patients to store and review various diabetes-related personal health information (such as blood pressure, weight, exercise, diet, medications, and symptoms) appears to be another popular feature in many apps. Furthermore, the personalized notification is included in some apps.

Some of the studied apps provide diabetes-related analytical result and decision-making support for users. Various functionalities such as providing guidelines regarding the treatment and management of diabetes, multimodal educational materials (eg, videos, forums), and PHR synchronization are also included in some apps to examine patients’ lifestyle.

A less presented, but interesting, functionality is the social media support, such as the option to email, text message, or any type of communication between stakeholders (eg, patients, service providers, patent’s relatives, friends). Few apps also provide data connectivity, which allows for end-to-end data transmission. Table 2 shows the presence of functionalities in the studied apps (N=78).

**Table 2:** Percentage of studied apps having different features (N=78).

Features	n	%
Blood glucose	78	100
Blood pressure	58	74
Weight	45	58
Notification and alert	42	54
Decision supporting	35	45
Record of exercise	34	44
Diet status	33	42
Social media	30	38
Record of medications	28	36
Diabetes-related education material	19	24
Data evaluation and analysis method	14	18
Data synchronization to personal health record	12	15
Record of symptoms	7	9
Bluetooth	10	13

###  Patents

A total of 39 identified patents have been downloaded and their functionalities were studied. The described solutions in most of the studied patents are designed as an end-to-end SMBG system. Some of them even go beyond the PHR server and interact with HIS. The personal information (eg, patient’s medical records, health insurance information) is also recorded in some of the studied patents.

The studied patents have many functionalities in common. In addition, many functionalities present in the studied apps are also included in the studied patents, such as decision-supporting information, notification and alert, analysis, record of personal health information, and social media. Table 3 shows the most dominating features and the percentage of the studied patents (N=39) that have these tools.

**Table 3:** Percentage of the studied patents having different features (N=39).

Features	n	%
Mobile terminal	33	85
Server	27	69
Bluetooth	9	23
Decision supporting	10	26
Notification and alert	7	18
Data evaluation and analysis method	6	15
Social media	5	13
Record of personal information	5	13
Record of personal health information	4	10
Interface to the hospital information system	7	18
Diabetes-related educational material	6	15

## Discussion

### Comparison of Functionalities Between Apps and Standards

All the functionalities related to data summary are shown in Tables 1 and 2, which indicate good conformance of the studied apps to the aforementioned referenced standards. However, for some other types of functionalities, the situation is different. The details are discussed in the following sections.

#### Connectivity

The connectivity is a key component in an end-to-end SMBG system. According to the results presented in Table 2, it can be seen that only 13% (10/78) of the studied apps have connectivity; however, all of these use Bluetooth [43]. The deployment of traditional manual input may have negative impacts, such as poor usability, wrong data entry, and barriers of integration. Most apps have no connectivity to the measuring instruments; a possible reason for this may be the obsolete mind-set of domestic app developers and instrument manufacturers. When building their own products, their design logic is purely app centric or device centric, rather than having a holistic vision established over an interconnected infrastructure. By contrast, as leading tech companies, Google recently released Google Fit [44] and Apple released Health Kit [45]; both of them leverage connectivity technologies to simplify or automate the heath data-collection process. It is quite obvious that market players are keen on adopting such functionality in this domain. We believe a similar trend will appear in the future in the Chinese market, but the questions are just “when” and “how.”

#### End-to-End Data Synchronization Function

As shown in Table 2, only 15% (12/78) of the apps have the end-to-end data synchronization function that allows the data generated by devices to be transmitted and stored in the PHR database, possibly via certain gateway device. Among these 15% apps (12/78), only 1 provides synchronization with a third-party platform (the Microsoft Health Vault [46]); all others connect to their own proprietary health portal.

Despite the benefits of PHR synchronization reported in some studies [47,48], the adoption rate of such functionality is less than what we initially expected, partially because of end-to-end usability issues. Our interpretation of the possible reason is that gateway device vendors are quite dominant in the current Chinese ICT market (including the ecosystem of ICT-powered SMBG), and the app vendors mainly focus on the data synchronization with the gateway device, rather than on the cloud platform. Such strategy helps them to quickly build a relationship with gateway device vendors, and start to share the revenue in the near future; however, this may not be an optimal strategy in the long run, because the service providers are likely to be situated behind the cloud. Without the service providers, the ICT-powered SMBG implementations will become much less attractive to the users. To improve the aforementioned usability issue, joint effort from all the stakeholders (operators, integrators, medical device manufacturers, app developers, health care providers, etc) must be in place to establish a data synchronization channel.

#### Standardized Data Structure

Among all the portals we have studied, the core diabetes-related data structures are proprietary. None of them is consistent with the data structure suggested by the aforementioned referenced data standards. In this situation, such portals could not be integrated into arbitrary HISs in a massive scale, and thus, these data cannot be exchanged. As a result, most of the portals have only minimal dataset, which is insufficient to support the decision making of clinicians. Luckily, the MoH has noticed this gap, and has begun to tackle it. We expect to see the first wave of standards released within 3-4 years.

#### Social Media

Findings from this study suggest that only 38% (30/78; Table 2) of apps contain social media functionality. Most apps that include social media support only provide data-sharing interface to the well-known Chinese social networking services such as WeChat [49] and Weibo [50]. It is hard to see any other interaction between the studied SMBG apps and the linked social networking services. We have also noticed that it is not easy to share graphs and data via the studied SMBG apps with friends or relatives in social networks.

However, we still consider the availability of this functionality as a positive sign, because the psychological motivation coming from others is a good stimulator for patient’s self-management. For example, Chen [51] reported the importance of social aspects and experience sharing among people with diabetes. It is interesting to find that the social media functionality available in these studied apps is not listed in Table 1, that is, it is not included in the current clinical guidelines. We are eager to see how and when such innovative functionality coming from the ICT world could someday result in a change in the traditional clinical world.

### Analysis of Patents

According to our study, glucose measuring module, mobile terminal, and remote server contained in most patents constitute the general technical architecture of the end-to-end and closed-loop SMBG systems.

Because inventors often focus sharply on the innovative contents, most patents in our study did not necessarily contain all the SMBG functions defined by referenced standards. The claims of most patents just introduce some common functionalities of the SMBG system. The social media functionality is also found in the contents of studied patents, which is not included in Table 1.

### Comparison Between the Studied Patents and Apps

**Figure 6:**
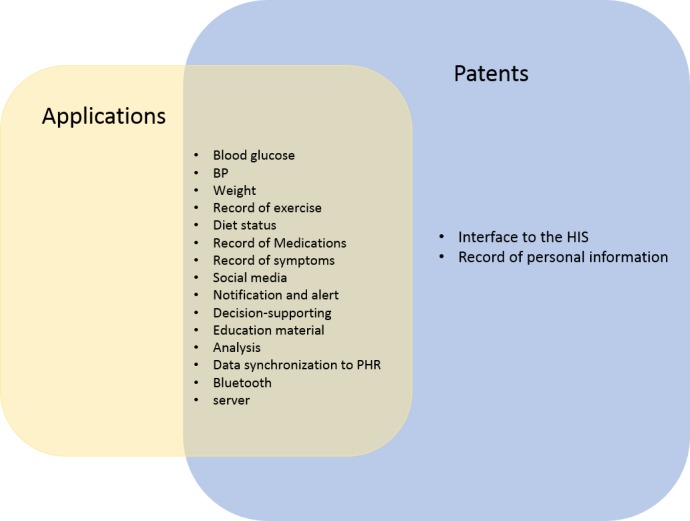
Common features identified in applications and in patents.

Although many of the functionalities identified in this study were found to be common in app and patents, functionalities such as “interface to the HIS” and “recording personal information” have been found to be missing in many apps. Figure 6 shows the distribution of functionalities in the studied apps and patents.

This finding suggests that when writing the patents, the big picture of end-to-end SMBG system is considered; however, when it comes to implementation, only practically feasible requirements are considered due to many practical difficulties (eg, the connectivity interface between the hospital and apps). At present, regulators in both MoH and CFDA have not yet realized the potential operational and security risks caused by ICT-powered SMBG systems, and thus, no regulatory policy includes any holistic requirement taking the end-to-end scenario into consideration. However, by judging the recent policy developments in the European Union and the United States, we believe the situation will be changed soon in China.

Compared with the patents (N=33), less portion of apps (n=10) contains the data transmission function. This is because writing a patent does not require any actual implementation of functionality. By contrast, app developers have to prioritize their tasks according to reality. They tend to implement any functionality that is feasible to be implemented at that moment. In particular, for implementing the data transmission function, the developers may confront difficulties in engaging its supplier, interfacing with peer device, and the corresponding development cycle might be lengthy and costly.

### Limitations

Many of the apps found in the online stores were not available for installation. As a result, some of the functionalities were recorded only from the verbal description or from published articles. Often there are discrepancies between the text description and the actual features, and some functionalities are not apparent until the app is installed and tested.

Another limitation may be that only patents in Chinese were included in this study. In fact, many scholars and manufactures in China file their patents internationally. These international patents have not been searched in our study.

This study only focused on the functionality of apps and patents in Chinese. The clinical efficiency and cost efficiency, however, are not evaluated. We will address these limitations in our future study.

###  Conclusions

In this article, we reviewed the ICT-powered SMBG systems in PR China. We reviewed 289 apps and 8070 patents. Eventually, 78 apps and 39 patents were analyzed and compared in multiple aspects, including innovative functionalities, defects, prospects, conformance to data standards, conformance to clinical guidelines, etc.

The main finding is that the apps still have some missing links, including the connectivity, the end-to-end data synchronization function, and the standardized data structure. The other major finding of this study is that the glucose measuring module, mobile terminal, and remote server contained in most patents constitute the general technical architecture of the end-to-end and closed-loop SMBG systems. The other difference between the patents and apps is that a couple of features—“interface to the HIS” and “record of personal information”—mentioned in the patents do not appear in the apps. It is also interesting to find that the social media feature that the apps and patents have is not included in the standard requirements list. In the future, the clinical guidelines can be further developed to leverage new features provided by ICT-powered SMBG apps.

The findings provide insights into the design and development of the ICT-powered diabetes self-management system to assist patients suffering from diabetes. Hence, to be able to come up with a good design of a diabetes support tool, all the aforementioned features have to be incorporated into a single app for good monitoring, better follow-up by health care professionals, and better lifestyle management for patients with diabetes.

## Appendix

Multimedia Appendix 1 Diabetes self-management applications list.

Multimedia Appendix 2 Diabetes self-management patents list.

## Abbreviations

BGM: blood glucose monitor
CFDA: China Food and Drug Administration
CGM: continuous glucose monitor
HbA1c: glycosylated hemoglobin
HIS: hospital information system
ICT: information and communication technology
IEEE: Institute of Electrical and Electronics Engineers
MoH: Ministry of Health
PHR: Personal health record
SMBG: self-monitoring of blood glucose
